# Fetal Lymphoid Organ Immune Responses to Transient and Persistent Infection with Bovine Viral Diarrhea Virus

**DOI:** 10.3390/v12080816

**Published:** 2020-07-28

**Authors:** Katie J. Knapek, Hanah M. Georges, Hana Van Campen, Jeanette V. Bishop, Helle Bielefeldt-Ohmann, Natalia P. Smirnova, Thomas R. Hansen

**Affiliations:** 1 Department of Biomedical Sciences, Colorado State University, Fort Collins, CO 80523, USA; kjknapek@central.uh.edu (K.J.K.); h.georges@colostate.edu (H.M.G.); hana.van_campen@colostate.edu (H.V.C.); jeanette.bishop@colostate.edu (J.V.B.); natalia.smirnova@sidelinesoft.com (N.P.S.); 2 Department of Microbiology, Immunology and Pathology, Colorado State University, Fort Collins, CO 80523, USA; 3 Australian Infectious Diseases Research Centre and School of Veterinary Science, The University of Queensland, St. Lucia, QLD 4072, Australia; h.bielefeldtohmann1@uq.edu.au

**Keywords:** bovine viral diarrhea virus, fetus, thymus, immune response

## Abstract

Bovine Viral Diarrhea Virus (BVDV) fetal infections occur in two forms; persistent infection (PI) or transient infection (TI), depending on what stage of gestation the fetus is infected. Examination of lymphoid organs from both PI and TI fetuses reveals drastically different fetal responses, dependent upon the developmental stage of the fetal immune system. Total RNA was extracted from the thymuses and spleens of uninfected control, PI, and TI fetuses collected on day 190 of gestation to test the hypothesis that BVDV infection impairs the innate and adaptive immune response in the fetal thymus and spleen of both infection types. Transcripts of genes representing the innate immune response and adaptive immune response genes were assayed by Reverse Transcription quatitative PCR (RT-qPCR) (2^−ΔΔCq^; fold change). Genes of the innate immune response, interferon (IFN) inducible genes, antigen presentation to lymphocytes, and activation of B cells were downregulated in day 190 fetal PI thymuses compared to controls. In contrast, innate immune response genes were upregulated in TI fetal thymuses compared to controls and tended to be upregulated in TI fetal spleens. Genes associated with the innate immune system were not different in PI fetal spleens; however, adaptive immune system genes were downregulated, indicating that PI fetal BVDV infection has profound inhibitory effects on the expression of genes involved in the innate and adaptive immune response. The downregulation of these genes in lymphocytes and antigen-presenting cells in the developing thymus and spleen may explain the incomplete clearance of BVDV and the persistence of the virus in PI animals while the upregulation of the TI innate immune response indicates a more mature immune system, able to clear the virus.

## 1. Introduction

Bovine viral diarrhea viruses (BVDV) cause significant economic losses in all sectors of cattle production worldwide [1,2,3,4]. BVDVs are small single-stranded RNA viruses belonging to the Pestivirus genus in the family Flaviviridae [5]. Isolates of BVDV are classified into two genotypes, type 1 and type 2, with several subtypes and two biotypes: cytopathic (cp) and noncytopathic (ncp) [6]. Acute BVDV infection of immunocompetent cattle results in diverse clinical presentations, including subclinical infection, fever, nasal and/or ocular discharge, pneumonia, severe systemic disease, hemorrhage, and peracute death [7]. Importantly, BVDV infection of pregnant cattle results in fetal infection and reproductive losses, including early embryonic death, abortion, and stillbirth [8,9,10,11]. If maternal infection with ncp BVDV occurs prior to 125 days of gestation, the fetus becomes persistently infected (PI) with the virus and is born without BVDV-specific antibodies [12,13]. PI animals will shed BVDV throughout life, serving as the main source of infection for other cattle. BVDV infection of pregnant cows after 150 days of gestation results in a transient infection (TI) of the fetus. These TI calves are born with BVDV-specific antibodies indicative of a functional adaptive immune response and clearance of the virus [9,14,15]. Virus-specific immune responses in the bovine fetus develops between days 125 and 150; therefore, BVDV infection during this time may result in either a PI or TI, depending on the individual fetuses [16]. The differences in the outcomes of PI and TI fetal infections have been attributed to the maturation and function of the fetal immune system at the time of infection [17].

At least two explanations have been tendered to explain the persistence of BVDV in PI cattle. First, ncp BVDV has been shown to inhibit type I interferon (IFN) induction in vitro through the actions of virally encoded RNase (E^rns^) and the N-terminal protease (N^pro^) (reviewed in [18,19,20,21]). The latter N^pro^ degrades *interferon regulatory factor 3* (IRF3), thus inhibiting the transcription of IFNB and its antiviral activity in adjacent cells [22,23,24]. E^rns^ binds to and degrades double-stranded RNA (dsRNA), preventing its binding to cells and induction of IFNs [25]. Inhibition of the IFN response by N^pro^ and E^rns^ allows BVDV to persist and replicate in cells. Conversely, in an in vivo model, mutations of these two viral genes affecting their functions negate persistent infection [26]. In another in vivo model using BVDV strain Pe515nc, fetuses were inoculated in utero (amniotic fluid) and collected on days 3, 5, and 7 post-inoculation [27]. BVDV was confirmed in fetal tissues; however, an innate immune response was not seen, suggesting an inhibition of the fetal innate immune response to BVDV [27]. A second explanation of BVDV persistence in fetal infections might be that BVDV does not inhibit the IFN response, but instead, another unknown mechanism is responsible for persistence. Previously, we observed that bovine fetuses whose dams were inoculated with ncp BVDV 96B2222 on day 75 of gestation had a peak in BVDV RNA in fetal blood on day 97 of gestation, 21 days post-maternal inoculation (dpmi), followed by a 10-fold decrease in viral RNA on days 192 and 245 of gestation, suggesting partial viral clearance by an active immune response in the PI fetus, or possibly a decrease in infected cell types/numbers [28,29,30]. Interferon stimulated genes (ISG), such as *ISG15*, *protein kinase RNA-activated* (*PKR*), as well as the RNA helicases: *DEAD Box Protein 58* (*DDX58*), also known as *retinoic acid-inducible gene* (*RIGI), melanoma differentiation-associated protein 5* (*MDA5*); and *DExH-box helicase 58* (*DHX58*) were also shown to be chronically upregulated in the PI animal postnatally [31]. These results indicate that PI fetuses and placenta respond to BVDV with an innate immune response, albeit somewhat reduced compared to TI fetuses [28,29,31]. These findings indicate that the fetus responds to ncp BVDV infection with an innate immune response, much like the innate response to ncp BVDV infection in postnatal calves shown by Palomares et al. (2013); hence, inhibition of the innate immune response by viral proteins does not entirely explain viral persistence in vivo [32]. Another in vivo postnatal model used BVDV strain 11,249, which was previously shown to inhibit the IFN response in vitro [33]. The study revealed that in vivo, this BVDV strain does induce IFN responses [33]. The stimulation of an IFN response to BVDV has been exhibited in in vitro studies, in vivo fetal infection studies, and in vivo postnatal infections. Differences in these theories could be explained by differences in BVDV strains used and/or differences in experimental models. It is important to examine these differences and theories when considering the results of the present study.

An additional explanation for viral persistence is that the presence of BVDV during the development of the T cell repertoire permits its antigens to be accepted as “self” antigens, resulting in a state referred to as immunotolerance in which elements of the adaptive immune system do not respond to viral antigens and do not clear the virus from fetal tissues [34]. We hypothesize that ncp BVDV fetal infection early in gestation (<125 days) interferes with T cell development in the bovine fetal thymus during a critical period in which T cells are selected based on their recognition of self-antigens, affecting T cell response to BVDV in the spleen, resulting in immunotolerance to the virus and persistent infection in the calf. BVDV fetal infection later in gestation (>150 days) results in an active innate immune response, which clears the virus infection and causes a delay in antigen-specific T and B cell responses. To examine the effect of ncp BVDV infection on the bovine fetal thymus and spleen, transcripts of genes representative of the innate and adaptive immune response pathways were compared with control fetuses collected on day 190 of gestation.

## 2. Materials and Methods

### 2.1. Animals

Animal experiments were performed as previously described [28,34]. All animal experiments were approved by the Institutional Animal Care and Use Committees at the University of Wyoming, approval 05-265A-02 (19/10/2005) and 08-16A-01 (11/01/09). BVDV antigen negative and seronegative yearling Hereford heifers were synchronized for estrus (ovulation) and artificially inseminated with BVDV-free semen. Pregnancy was confirmed by ultrasound examination on days 35 to 40 and on day 70 of gestation.

### 2.2. Experimental Design: BVDV Inoculation and Fetal Collections

A power analysis was performed, and a group of 6 animals per treatment group (control, TI, and PI) was determined to be appropriate for a power of 1. Therefore, 18 unvaccinated pregnant heifers were randomly placed into treatment groups and inoculated intranasally with 2 mL culture media to generate sham-treated controls (*n* = 6) or with media containing 4.4 log_10_ TCID_50_/_mL_ of ncp BVDV2 strain 96B2222 on day 75 of gestation to generate PI (*n* = 6) fetuses and on day 175 to generate TI (*n* = 6) fetuses [34]. Treatment groups were kept in widely separated pens and fed at the end of feeding rounds to minimize viral transmission. To capture the fetal immune response during maternal seroconversion (in the PI group), day 190 of gestation was chosen for fetal collections. Eighteen fetuses were collected by Cesarean section and necropsied on day 190 of gestation. Samples of fetal thymuses and spleens were frozen in liquid nitrogen and stored at −80 °C.

### 2.3. RNA Extraction and RT-qPCR

Total RNA from 70 mg of frozen thymus and 50 mg of frozen spleen was isolated using TRIzol reagent according to the manufacturer’s instructions (Invitrogen ThermoFisher, Rockford, IL, USA). The isolated RNA was treated with DNase I (Qiagen, Germantown, MD, USA) and purified using the RNeasy MiniElute Cleanup Kit (Qiagen, Germantown, MD, USA). RNA concentration and 260/280 and 260/230 ratios were measured using the NanoDrop 1000 Spectrophotometer (ThermoScientific, Rockford, IL, USA). The primer sequences and gene accession numbers are listed in Table 1. One µg of RNA was reverse transcribed to synthesize cDNA using iScript™ Reverse Transcription Supermix (Bio-Rad, Hercules, CA, USA). Reverse transcription quantitative polymerase chain reaction (RT-qPCR) was performed with iQ™ SYBR^®^ Green Supermix (Bio-Rad, Hercules, CA, USA). Each cDNA reaction was diluted (1:5 for thymus and 1:10 for spleen) with RNase-free water. Primers were used at 3 µM concentration. Each sample was assayed in duplicate wells on a 384-well plate. Four biological replicates of RT-qPCR plates were performed at one cycle of 95 °C for 3 min, 40 cycles of 95 °C for 30 s, 58 °C for 30 s, and 72 °C for 15 s with a final 5 min elongation in a LightCycler-480 Instrument (Roche, Basel, Switzerland). Upon completion of RT-qPCR, melting curve analysis was performed to assess the quality of amplification.

### 2.4. RT-qPCR Targets and Validation

RT-qPCR targets for each tissue were chosen based on current knowledge and available tissue. Targets shared between the thymus and spleen were chosen to understand basic changes in both the innate and adaptive branches of the immune system. For the innate immune branch, targets chosen were *DDX58*, *NFKB*, *IRF7*, *IFNB*, and *ISG15*. For the adaptive branch, targets chosen were *PSMB9*, *IFI30*, *CD4*, *CD8A*, *CD8B*, and *CD79B*. Thymic tissue samples were more abundant than the spleen, allowing for additional targets to be studied in the thymus; *STAT1*, *IFI6*, *CXCL10*, *CXCL16*, *CXCR6*, *TAP1*, *B2M*, *CIITA*, and *CD46*. These results can be found in Table A1. A previous study which used a microarray to study BVDV-infected spleens indicated *STAT4* and *PSMB8* as targets of interest specific for splenic samples [35]. Therefore, both *STAT4* and *PSMB8* were additional targets for splenic samples in this study. BVDV viral RNA was also targeted to confirm tissue infection in TI and PI fetuses. These results can also be found in Table A1. Standard RT-qPCR primers were validated and evaluated based on MIQE guidelines [36].

### 2.5. Morphogenesis of Thymus during Bovine Fetal Development and Effect of In Utero BVDV Infection

Fetal thymus and spleen samples were fixed for 48 h in 10% neutral-buffered formalin, transferred into 70% ethanol, and paraffin-embedded. Four to 5 µm sections were cut and stained with eosin and hematoxylin (H&E). Microscopic assessment was performed with special attention to the components of the reticulo-endothelial network, myeloid, and lymphoid cells, including where the latter two cell types appear in the organ primordium. The relative cortical-to-medulla ratio was assessed, including full encirclement of the medulla by cortical thymocytes. Additionally, attention was paid to myoid cells and Hassall’s corpuscles in terms of appearance, frequency, and size.

### 2.6. Statistical Analysis

RT-qPCR data are presented according to MIQE guidelines and statistically analyzed using the 2^−ΔΔCq^ method [37]. Briefly, the Δ quantification cycle (C_q_; also known as C_t_) was calculated by subtracting the mean C_q_ of the reference gene (18S rRNA), within the treatment group, from the C_q_ of the target gene (same treatment group as the reference). The average of the Δ C_q_ for controls for the target gene were then subtracted from each infection treatment Δ C_q_, for the same target gene as controls, to calculate the fold change (2^−ΔΔCq^). Statistical analysis of data obtained by RT-qPCR (2^−ΔΔCq^) was performed in GraphPad Prism 8 (GraphPad Software, San Diego, CA, USA). The data were checked for normality using Shapiro-Wilks test; normally distributed data were analyzed with a one-way ANOVA and Dunnett’s multiple comparisons test, while non-normally distributed data were analyzed with a Kruskal-Wallis test and Dunn’s multiple comparisons test. Significant differences were at *p* < 0.05 and tendencies/trends were at *p* < 0.10. Data are presented as the mean ± SEM. Graphical data represents 2^−ΔΔCq^ of which statistical analysis was run. In the text, fold change is presented for ease of understanding up- and down-regulation. For 2^−ΔΔCq^ values > 1, fold changes were reported as averaged 2^−ΔΔCq^ values. For 2^−ΔΔCq^ values between 0 and 1 (down regulation), the negative inverse (−1/(2^−ΔΔCq^)) was calculated and reported as fold change.

## 3. Results

### 3.1. Detection of BVDV RNA Expression in Thymus and Spleen and BVDV Receptor CD46 in Fetal Thymuses

**Figure 1:**
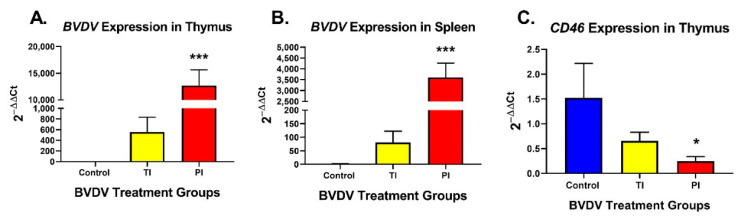
Bovine Viral Diarrhea Virus (BVDV) expression in (**A**) thymus and (**B**) spleen samples (Control, TI, and PI). Expression of BVDV was significantly upregulated in PIs compared to controls in both thymic and splenic samples. Expression of BVDV in TIs was higher than controls, but not significant due to individual variation. (**C**) BVDV receptor CD46 expression in thymus (Control, TI, and PI). *CD46* expression was significantly down-regulated in PI thymuses. Control: Uninfected, blue; TI: Transiently Infected, yellow; PI: Persistently Infected, red. * *p* < 0.05, *** *p* < 0.001.

BVDV RNA was not detected in any of the control fetal thymus RNA samples and was present in 6 of 6 PI and 5 of 6 TI fetal thymic RNA samples with PI thymuses having 9.7- to 68.8-fold higher BVDV RNA concentrations compared to TI thymuses (Figure 1A). PI fetal spleens had a significantly increased amount of BVDV RNA (*p* < 0.001, 2^−ΔΔCq^ = 3037-fold greater) compared to controls (2^−ΔΔCq^ = 1.0) and very low amounts of BVDV RNA in TI fetal spleens (Figure 1B). The concentration of BVDV receptor *CD46* mRNA in PI fetal thymuses was decreased 6.2-fold (*p* < 0.001) and increased in TI fetal thymuses (*p* < 0.05) compared to controls on day 190 (Figure 1C).

### 3.2. Thymic Responses

#### 3.2.1. Innate Immune Responses in TI and PI Fetal Thymuses

**Figure 2:**
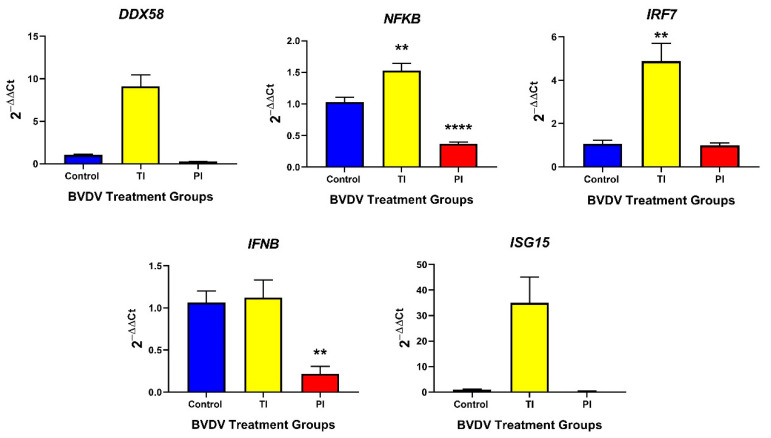
Control, TI, and PI thymus transcripts associated with the innate immune response. Interferon transcriptional regulators *NFKB* and *IRF7* were significantly upregulated in TI fetuses compared to controls. In PI fetal thymuses, only IFN transcriptional regulator *NFKB* and *IFNB* were down-regulated compared to control fetuses. TI: Transiently Infected, PI: Persistently Infected, ** *p* < 0.01, **** *p* < 0.0001.

In TI fetal thymuses, mRNA concentrations were significantly increased (*p* < 0.01) for the innate immune response genes *NFKB* (1.5-fold) and *IRF7* (4.5-fold) compared to controls on day 190 (15 dpmi). Neither *DDX58*, *IFNB*, nor *ISG15* were significantly different than the controls (Figure 2).

In the PI fetal thymuses, the mRNAs of the innate response genes *NFKB* (−2.8-fold) and *IFNB* (6.0-fold) were significantly downregulated in PI thymuses compared to controls on day 190 (*p* < 0.01) (Figure 2); whereas *DDX58*, *IRF7*, and *ISG15* concentrations remained at control levels and were not significantly different between control and PI thymuses. A trending decrease in the type I IFN stimulated transcripts *STAT1* (−22.7-fold; Table A1) was found in PI fetal thymuses compared to controls (*p* < 0.10), which was consistent with the observed downregulation of *IFNB*. Type II IFN stimulated transcripts, transitional responses from the innate to adaptive immune response, *IFI16* (−11.3-fold, trend *p* < 0.10) and *CXCL16* (−22.4-fold, *p* < 0.05) were similarly down-regulated (Table A1). *CXCR6* mRNA concentration was not different between control and PI fetal thymuses (Table A1).

#### 3.2.2. Adaptive Immune Responses in TI and PI Fetal Thymuses

**Figure 3:**
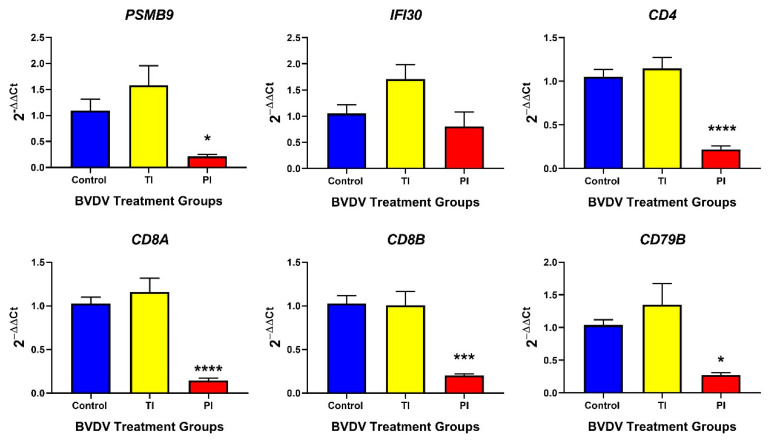
Thymic (Control, TI, and PI) transcript expression associated with the adaptive immune response. No significant differences were found in TI fetal thymuses compared to controls. However, all adaptive genes, with the exception of *IFI30*, were significantly down-regulated in PI fetal thymuses compared to controls. TI: Transiently Infected, PI: Persistently Infected. * *p* < 0.05, *** *p* < 0.001, **** *p* < 0.0001.

In TI fetal thymuses, the expression of *MHC I* mRNA *PSMB9* (Figure 3), *TAP1* (Table A1), and *B2M* (Table A1) were not significantly different from controls. T cell markers *CD8A*, *CD8B*, and *CD4* and activated B cell marker *CD79B* mRNAs were not significantly different in TI fetal thymuses compared to controls.

The relative expression of MHC I antigen presentation pathway genes *PSMB9* (−5.2-fold)*, TAP1* (−5.5-fold)**, and *B2M* (−27.9-fold) transcripts were significantly decreased in PI fetal thymuses relative to controls on day 190 of gestation (*p* < 0.05) (Figure 3, Table A1 respectively). CTL co-receptors *CD8A* (−16.2-fold) and *CD8B* (−9.6-fold) mRNA concentrations were significantly decreased in PI fetal thymuses compared to the controls on day 190 of gestation (*p* < 0.05) (Figure 3). *CIITA* (−3.4-fold, trend *p* < 0.10) and *CD4* (−8.1-fold, *p* < 0.0001) mRNA associated with MHC II antigen presentation were significantly decreased in PI fetal thymuses on day 190 (Table A1 and Figure 3, respectively). Activated B cell marker, *CD79B*, mRNA concentration was significantly decreased (−3.8-fold) in PI fetal thymuses compared to control (*p* < 0.001) thymuses at day 190 of gestation (Figure 3).

#### 3.2.3. Morphogenesis and Histology of the Thymus during Bovine Fetal Development and the Effect of in Utero BVDV Infection

There were no discernible differences between the control or PI fetuses with regard to the thymus morphology at day 190. The morphology of the fetal thymus had attained almost a neonatal appearance, with the cortex having expanded to completely surround the medulla, and the cortico-medullary ratio being ≥2. The medulla contained large, often complex Hassall’s corpuscles, large numbers of myoid cells, and eosinophilic granulocytes. The latter could be seen throughout the medulla, but appeared in highest frequency in the cortico-medullary border zone. The myoid cells were large, round cells with a deeply eosinophilic cytoplasm, either homogenous or with finely fibrillar patterning. The almost euchromatic nucleus was round and centrally located, or more oval and acentric. A small amount of chromatin clumping along the nuclear membrane and occasionally a small nucleolus was present. These cells may, in some cases, be considered “single-cell” Hassall’s corpuscles.

### 3.3. Splenic Responses

#### 3.3.1. Innate Immune Responses in TI and PI Fetal Spleens

**Figure 4:**
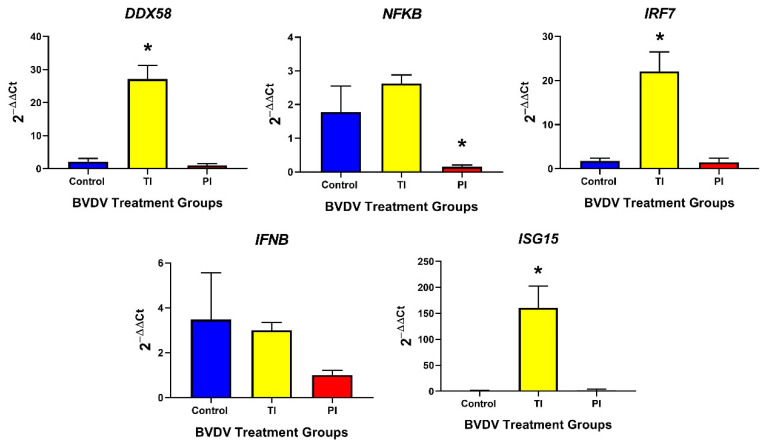
Spleen (Control, TI, and PI) expression of transcripts associated with the innate immune response. TI fetal spleens exhibited an upregulation of *DDX58*, *IRF7*, and *ISG15* compared to controls, indicating an active type I IFN response. In PI fetal spleens, only *NFKB* was downregulated compared to controls. TI: Transiently Infected, PI: Persistently Infected. * *p* < 0.05.

*DDX58* (25-fold; *p* < 0.05), IRF7 (22-fold; *p* < 0.05), and ISG15 (160-fold; *p* < 0.05) mRNA were upregulated in TI fetal spleens compared to controls, whereas *NFKB* and *IFNB* mRNA concentrations remained at basal levels and were not significantly different (Figure 4). The innate immune response in PI fetal spleens was similar to controls with only *NFKB* being significantly downregulated in PI fetal spleens compared to controls (0.1532-fold, *p* < 0.05, Figure 4).

#### 3.3.2. Adaptive Immune Responses in TI and PI Fetal Spleens

**Figure 5:**
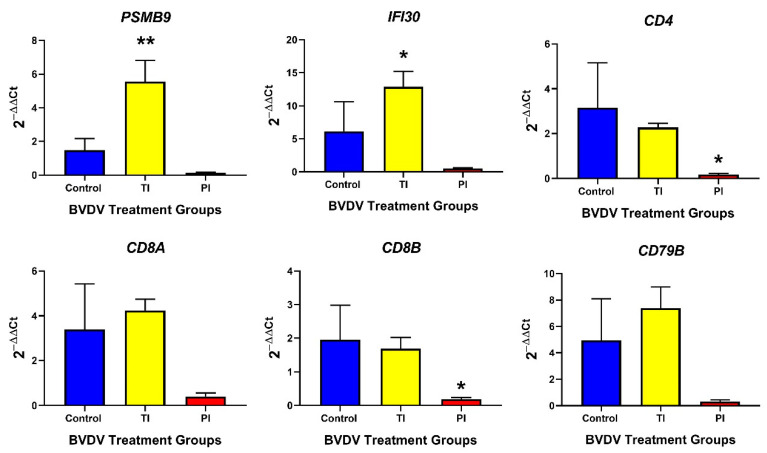
Splenic (Control, TI, and PI) expression of transcripts associated with the adaptive immune response. In TI fetal spleens, only *PSMB9* and *IFI30*, associated with antigen presentation, were significantly different to the controls. In PI fetal spleens, T cell markers *CD4* and *CD8A* were significantly downregulated compared to controls. TI: Transiently Infected, PI: Persistently Infected. * *p* < 0.05, ** *p* < 0.01.

Adaptive immune response genes, such as *STAT4* (Figure 5), *PSMB9* (MHC class I, Table A1), and *IFI30* (MHC class II; Figure 5) were significantly upregulated in TI spleens compared to controls (5.54-fold, *p* < 0.01; 12.9-fold, *p* < 0.05 respectively); however, *PSMB8* (MHC class I) was not significantly different between the two groups (Figure 5). T and B lymphocyte markers, *CD4, CD8A, CD8B*, and *CD79B*, were not different in TI compared to the control spleen (Figure 5).

In PI fetal spleens, *STAT4* (Table A1), *PSMB8/9* (Table A1 and Figure 5, respectively), and *IFI30* (Figure 5) were not differentially expressed compared to control fetal spleens. PI fetal spleens exhibited a downregulation of T lymphocyte markers mRNA (*CD4*, and *CD8B*) compared to controls (0.1465-fold, *p* < 0.5; 0.4005-fold, *p* < 0.05, respectively) (Figure 5) but T lymphocyte marker *CD8A* and B lymphocyte marker *CD79B* were not significantly different between the two groups.

## 4. Discussion

### 4.1. Transient Infection on Day 175 of Gestation Elicits an Immunocompetent Response in the Bovine Fetal Thymus and Spleen on Day 190

BVDV takes approximately 7–14 days to cross the placenta and infect the fetus following intranasal inoculation of the dam [38]. The TI fetal spleens and thymuses collected at day 190 of gestation, 15 days post-maternal infection were positive for BVDV RNA, indicating that the fetuses were infected for 1–7 days, which is sufficient time to have elicited an innate immune response in all TI fetuses and initial adaptive responses in some TI fetuses.

The rudimentary thymus is first detected around day 27 of gestation in ruminants with colonization and lymphoid development occurring at day 42 of gestation. Day 42 progenitor cells come from the fetal liver until the bone marrow develops and matures enough (around day 70) to take over as the primary source of thymocytes [39]. The bovine fetal thymus contains a discernable cortico-medullary delineation by day 42, Hassall’s corpuscles by day 65, and IgM positive cells by day 70 to 100 [40]. In addition to T lymphocytes and prothymocytes of T cell lineage, the postnatal thymus contains thymic epithelial cells, myoid cells, fibroblasts, dendritic cells, macrophages and miscellaneous neutrophils, eosinophils and B cells [41,42]. These cell types would be present in the thymus at the time of maternal inoculation with BVDV (75 dpmi and 175 dpmi) and subsequent fetal infection in this experimental model. In utero BVDV infections have been shown to result in thymic hypoplasia in bovine fetuses characterized by marked lymphocyte depletion in the cortex, accompanied by hypertrophy of the epithelial stroma and infiltration by monocytes and mature macrophages with BVDV antigens primarily localized to macrophages [43,44,45].

The innate immune gene transcripts, *NFKB*, and *IRF7* were significantly upregulated in TI fetal thymuses indicative of a robust activation of the innate immune response to BVDV, which agrees with previous findings [34,35,38]. Significant changes were not observed in adaptive response genes, including the T cell marker transcripts, *CD8A*, *CD8B*, *CD4*, or B cell activation marker *CD79B* compared to controls, as expected due to the short course between fetal infection and tissue collection. In addition, the thymus is a primary lymphoid organ responsible for the selection and maturation of T cells but with limited antigen-specific immune responses. The decreased BVDV RNA concentrations in TI thymuses compared to PI thymuses, however, is supportive evidence that TI fetuses were able to mount an effective immune reaction against the virus. Despite the ability of TI fetuses to clear the virus, BVDV fetal infection during day 175–190 of gestation occurs during a critical stage of thymus and spleen development, as well as T cell selection and maturation in the thymus. BVDV fetal infection during this stage of fetal development may alter the animal’s ability to fight other infections postnatally.

The spleen is a secondary lymphoid organ responsible for rapid immune responses to bloodborne pathogens like BVDV. During embryonic development, mesenchymal progenitor cells differentiate into marginal reticular cells, fibroblastic reticular cells, and follicular dendritic cells (reviewed in [46]). These cells will contribute to the spleen stroma and begin to form the white pulp, a lymphocyte-rich area which is the site of antigen-specific immune responses to blood-borne pathogens [46]. White pulp establishment occurs at approximately 15 weeks of gestation in humans, assumed to be a similar time point for cattle [40,46]. At this early time point, few B cells cluster around splenic arterioles, and 3 weeks later, are joined by T cells [46]. The T cells form the periarteriolar sheath (PALS), while the B cells form a follicle around the PALS [46]. Lymphocyte infiltration is believed to occur well after persistent infection with BVDV. However, TI fetuses were infected following the establishment of the white pulp and lymphocyte infiltration of the spleen. This may play a large role in the transient and persistent infection of BVDV in fetuses, as discussed later.

In TI fetal spleens, the cytoplasmic sensor mRNA *DDX58*, transcriptional factor *IRF7*, and interferon-stimulated gene *ISG15* were upregulated indicating the detection of BVDV RNA by the immune system of TI fetuses and activation of the innate immune response. Transcripts for genes associated with antigen presentation, *PSMB9* and *IFI30*, were upregulated in the TI fetal spleen, suggesting that the antigen processing mechanisms of cells had been stimulated. However, transcripts for T and B cell markers were not increased as viral peptide presentation in the context of MHC I and MHC II would not have stimulated the proliferation of T and B cells in the short time period between fetal infection and tissue collection. In addition, T and B cell activation may also have been delayed in the TI fetuses. BVDV is known to inhibit lymphocyte activation, resulting in delayed T and B cell responses during acute infections of postnatal animals [47,48]. The low amounts of BVDV RNA detected in the TI fetal spleen compared to PI fetal spleens suggest the innate immune mechanisms effectively inhibited viral replication prior to the elaboration of an adaptive immune response. Additionally, the fetal spleen is assumed to have both T and B cells on day 105 of gestation. It can be assumed these lymphocytes in the spleen, once activated, contribute to the clearance of the virus, seen in TI postnatal animals [9,15].

### 4.2. PI Thymus and Spleen Exhibit Diminished or Inhibited Immune Responses at Day 190 of Gestation

The mechanism of BVDV PI is controversial. Some of the first in vitro studies on BVDV infection concluded that BVDV infection blocked the host’s interferon response contributing to the persistence of BVDV in fetuses infected before day 120 of gestation [21,23,24]. In a paper published in 2001, the authors infected fetuses in utero (through amniotic fluid) on day 60 of gestation and collected fetuses 3, 5, and 7 days post-maternal inoculation [27]. It was concluded that ncp BVDV inhibits the fetal IFN response [27]. Subsequently, using a model of maternal infection, the fetal IFN response was stimulated by BVDV 22 days post-maternal infection [28,31,34,35,38]. However, it is important to note the differences in BVDV strains used and the differences in experimental models. This current study follows several others with similar experimental models and the same BVDV strain that have demonstrated an active innate immune response in PI fetuses soon after the establishment of fetal infection. The discussion below highlights these previous findings [28,31,34,35,38].

Previously, it was established that the early gestation bovine fetus (day 75) could respond to ncp BVDV infection with an innate immune response indicated by the upregulation of cytosolic dsRNA sensors in fetal blood and spleen, type I IFNs in cotyledons, and ISGs in fetal tissues, including the liver and spleen [28,31,38]. Moreover, the IFNG protein, an important bridge between the innate and the adaptive immune response, was detected in the amniotic fluid and fetal blood, and *IFNG* mRNA was upregulated in liver, spleen, and thymus of PI fetuses 14–22 dpmi (89–97 days of gestation) [34]. The activation of these genes corresponded with a reduction in BVDV titer in PI fetal blood at day 190 and 245, suggesting partial clearance of the virus by an active innate immune response; however, BVDV continued to be present in fetal tissues, albeit at a reduced level (reviewed in [14,28,34]). We hypothesized that the persistence of ncp BVDV was due to exhaustion of the initial innate immune response, and impaired steps in the adaptive immune response leading to incomplete viral clearance in the PI fetus. In support of this hypothesis, two innate immune response mRNAs, *NFKB* and *IFNB*, the bridging mRNA *CXCL16*, and the adaptive immune response mRNAs, *PSMB9, TAP1, B2M, CD8A, CD8B, CD4*, and *CD79B*, were found to be significantly decreased on day 190 in PI fetal thymuses compared to controls, and T cell marker mRNAs (*CD4* and *CD8B*) were significantly downregulated in the PI spleen. However, as discussed below, it is believed attenuation of the immune system may be caused by Treg development, instead of our original hypothesis of immune system exhaustion [35,49,50,51].

*DDX58* binds to and recognizes viral RNA in the innate immune system pathway. A reduction in *DDX58* concentrations could negatively impact the ability of fetal cells to respond to BVDV RNA and subsequently impair the induction of IFN antiviral activities. Persistent inhibition of NFKB has been associated with inappropriate immune cell development or delayed cell growth. For example, mice null for NFKB signaling molecules have impaired thymic medullary epithelial cell formation and autoimmune disease [52]. The transcription factor NFKB is both induced by type 1 IFNs and is a transcriptional regulator of cytokine expression, including IFNB and IFNG [53]. The decreased *IFNB* mRNA observed in the PI fetal thymus on day 190 coincided with the decreased expression of *NFKB* and the IFNI-inducible gene mRNAs, *ISG15* and *CXCL16*. Reduced *IFNB* mRNA may be explained by a paucity of dendritic cells in the PI fetal thymus at this stage of development; however, differences in the dendritic cell population between PI and control thymuses collected on day 190 were not observed histologically. IFI16 is a cytosolic sensor for viral RNA/DNA, which transcriptionally regulates the expression of type I IFNs in a positive manner [54]. The trended downregulation of *IFI16* observed in the PI thymus could explain the decreased expression of *IFNB*.

The PI fetal thymus exhibited differences in the innate immune response genes at day 190 compared to those previously observed in the fetal spleen and blood. *DDX58, MDA5*, and *ISG15* were previously observed to be increased in PI fetal blood from day 97 to day 192, and *ISG15* was increased in the PI fetal spleen, bone marrow, caruncle, and cotyledon, reflecting an active innate response in these tissues [28,31]. In contrast, *NFKB* and *IFNB* were significantly downregulated in PI fetal thymuses on day 190, but only *NFKB* was downregulated in spleens. A previous study by our group indicated a significant down-regulation of *IFNB* in day 190 PI fetal spleens, but no significant change in *NFKB* [35]. These studies were independent, and although results differ slightly, they both indicate an inhibition of the innate immune response 115 days post-maternal infection. Regulatory T cells (Tregs) originate in the thymus during fetal development and create tolerance of self in the developing fetus to prevent autoimmune disorders [49,50]. Due to the fetal BVDV infection occurring early in fetal development, prior to lymphocyte infiltration in the spleen, it is possible the Treg cells are creating tolerance to the BVDV viral antigen, allowing the virus to continue replication without an immune response from the host [49,50]. The development of Tregs in the thymus could be contributing to the difference in immune gene expression between the thymus and spleen [49,50]. On day 190 of gestation in PI animals, Tregs may be inhibiting an immune response in the thymus first before the Treg cells migrate to the periphery and reach the spleen.

Thymic concentrations of adaptive immune response transcripts, such as MHC I antigen presentation (*PSMB9, TAP1*, and *B2M*), CTL co-receptors *CD8A* and *CD8B*, and T cell marker CD4 were also decreased in PI fetal thymuses compared to controls consistent with an impaired adaptive immune response. The reduction in antigen presentation gene transcripts in PI fetal thymuses were associated with reduced *CD79B*, a component of antigen recognition and activation in B cells. In this study, *PSMB9* expression in PI thymuses was decreased at day 190 of gestation, suggesting a reduction in the normal function of the immunoproteasome [55]. Following proteolysis of viral proteins by the immunoproteasome, TAP1 transports peptides to the endoplasmic reticulum (ER) where peptides bind to MHC class I molecules for presentation on the cell membrane. Decreased *TAP1* concentrations could negatively impact antigen presentation to immune cells. In addition, *B2M* mRNA in PI thymuses was significantly decreased at day 190 compared to controls. The B2M protein assembles with the MHC I molecule in the ER to form a stable MHC I complex necessary for antigen binding. When B2M is not present, MHC I remains in the ER, and the MHC I complex is not expressed on the cell surface [56]. Decreased expression of *B2M* would result in additional dysfunction of the endogenous viral antigen presentation mechanism. In PI fetal spleens, *PSMB9* and *IFI30* were not significantly different compared to controls. This contradicts a previous study in which PSMB9 and IFI30 were significantly downregulated in day 190 PI spleens compared to controls [35]. This difference between the studies reflect increased individual variability between controls in the present study or possibly due to slight variation in infection/fetal collection times. The difference in the expression of antigen presentation mRNAs between the spleen and thymus may be indicative of APC and lymphocyte migration from the primary lymphoid organ, that is, the thymus, to secondary lymphoid organs, such as the spleen. In secondary lymphoid organs, such as the spleen, APCs reside and actively survey the blood and lymph for antigens; thus, the secondary lymphoid organs will have higher gene expression of mature APCs and lymphocyte markers [57].

Cytotoxic T cells recognize peptides presented in the context of the MHC I and B2M complex on target cells by the T cell receptor (TCR) and co-receptor, CD8. Antigen recognition by the CD8-TCR complex triggers the release of perforin, granzymes and cytokines from the lytic granules of CTL. *CD8A* and *CD8B* mRNA concentrations in PI fetal thymuses and *CD8B* in spleens were significantly decreased at day 190, suggesting either a reduced number of CTLs or decreased CD8A/B expressed per cell. Similarly, *CD4*, the co-receptor of the T cell receptor on T helper cells, mRNA was decreased in PI fetal thymuses and spleens at day 190 compared to controls and may reflect a decrease in T helper cells or the number of co-receptors per T helper cell. Supporting the results in a previous study, a reduction in BVDV-specific CTLs, T helper cells, or in antigen recognition would negatively impact the clearance of BVDV-infected cells in the thymus, spleen, and other fetal tissues, thus contributing to viral persistence [35].

The B-cell antigen receptor complex-associated protein Beta chain, *CD79B*, is part of the B lymphocyte antigen receptor complex and is required for T cell-dependent activation of B cells. In cooperation with CD79A, CD79B activates B cells through the interaction with CD4 T cells. After this interaction, B cells then have the capability to proliferate, secrete antibodies, cytokines, and chemokines, and initiate memory cell formation [58]. CD79B gene expression was decreased in PI thymuses on day 190 compared to controls, but were not downregulated in PI fetal spleens. However, a previous study by our group did find a decrease in CD79B expression in PI fetal spleens [35]. This difference may be due to individual variation of animals in the current study or slight differences in infection/collection timing. A sustained decrease in CD79B expression could result in the depletion of B cells later in gestation or postnatally, contributing to the overall deficit in adaptive immunity, failed viral clearance, and BVDV persistence. The outcome would be apparent immunotolerance to BVDV and the inability of PI animals to combat secondary viral and bacterial infections, such as bovine respiratory disease in postnatal life.

CD46 has been shown to be a receptor for BVDV entry [59,60]. CD46 is a regulator of complement activation through its interaction with plasma serine protease factor I, which cleaves complement factors C3b and C4b to prevent cell injury from complement attack. The concentration of *CD46* was significantly decreased in PI fetal thymuses compared to controls at day 190. The reduced concentration of *CD46* in the PI thymuses may reflect a general diminishment of constitutively expressed cellular mRNAs due to viral mechanisms that usurp cellular transcriptional machinery in favor of the production of viral proteins [61]. Alternatively, expression of the BVDV receptor might be inhibited in response to the binding of BVDV to its receptor and the presence of high viral titers during persistent infection. The downregulation of *CD46* in PI thymuses might also reduce the regulatory activity of CD46 on the complement system and increase the vulnerability of host cells to complement-mediated injury. A soluble form of CD46 which neutralizes BVDV in vitro is present in adult bovine plasma and may play a role in viral clearance in postnatal acute infections. However, soluble CD46 is not found in fetal or neonatal serum, and is therefore unlikely to impact PI fetal BVDV infections [62].

The comparison of TI and PI fetal immune responses in developing lymphoid organs has not been previously examined in experimental settings. Unfortunately, bovine-specific antibodies for the genes of interest are unavailable, and limited this study to RNA expression only. The inability to obtain protein data does limit interpretation. However, the results of this transcriptome study exhibit a drastic difference in immune response competence and maturity between PI and TI fetuses. The upregulation of the innate immune response of TI lymphoid organs, along with the inability of BVDV levels to be significantly different than controls indicate that the TI fetus is effectively fighting the virus, unlike the PI fetus. The timepoint of TI fetal collections may have been too early to capture the activation of the adaptive immune response, or the virus is possibly able to temporarily inhibit the adaptive immune response. It is further hypothesized that although the TI fetuses are able to respond appropriately to the virus, this viral insult during fetal development epigenetically alters the TI animals’ DNA and causes further immune-related issues postnatally. This will require further studies postnatally.

## 5. Conclusions

**Figure 6:**
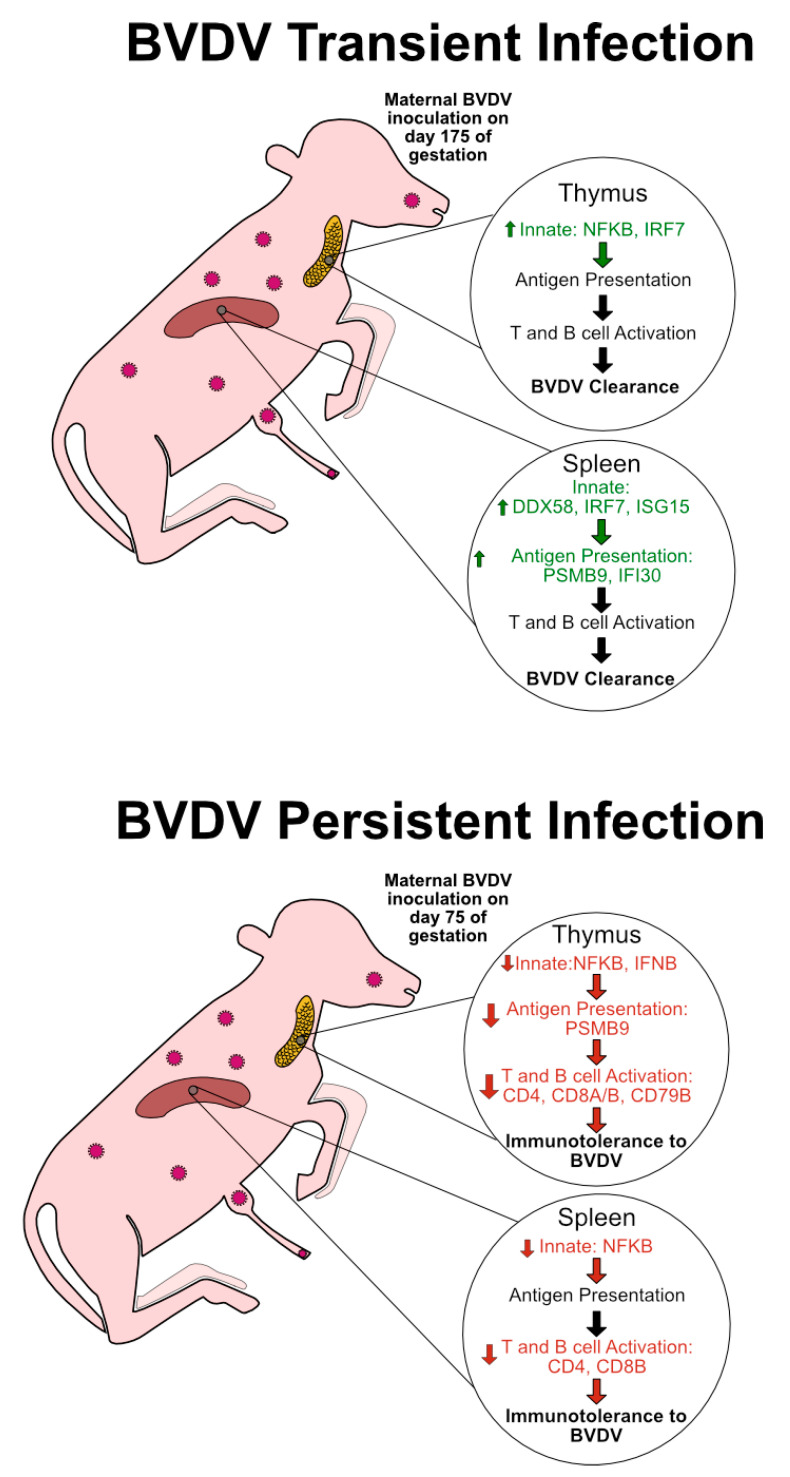
Graphic summarizing significant changes in fetal immune responses based on type of infection and tissue, as interpreted by RT-qPCR analysis. BVDV transient infection represents a fetal infection after gestational day 160 in which the fetus is able to clear the virus. Trends include an upregulation in genes associated with the innate immune response. Day 190 of collections may have been too early to capture an adaptive immune response in TI fetuses. However, we hypothesize that these pathways contribute to BVDV clearance in TI animals. BVDV-persistent infection represents a fetal infection before gestational day 120 in which the fetus is unable to clear the virus. Trends include a drastic down-regulation of the innate and adaptive branches of the immune system, and may lead to immunotolerance of the fetus to BVDV. In future studies, TI BVDV clearance and PI immunotolerance will be studied further with protein targets and flow cytometry. Green text, up arrow left of text, and listed genes represent pathways and genes which were significantly upregulated, as determined by RT-qPCR. Red text, down arrow left of text, and listed genes represent pathways and genes which were significantly downregulated as determined by RT-qPCR.

Although animals infected with BVDV in utero may be born morphologically normal, ncp BVDV fetal infection has significant effects on the expression of innate and adaptive immune responses. In TI animals infected late in gestation, the immune system is developed enough to fight and clear the virus prior to or following parturition. We interpret these RT-qPCR data to reflect an active and upregulated innate immune response in TI thymuses and spleens, seen as an increase in type I IFN transcriptional regulators (Figure 6). In the case of PI fetuses, the virus infects the fetus prior to complete immune system development, ultimately causing drastic attenuation of the immune system, possibly due to the fetus identifying the virus as “self” (Figure 6). Specifically, the downregulation of genes, including type I IFN transcriptional regulators, antigen presentation, and T cell markers in PI fetal tissues was found. These trends in TI and PI fetuses were seen in both the thymic and splenic tissues, suggesting that these changes occur simultaneously in tissues critical for immune system education and development (Figure 6). These types of changes are believed to affect the infected animals postnatally, possibly through epigenetic changes.

## Figures and Tables

**Table 1:** Primers utilized for RT-qPCR.

Gene	Sequence	Accession	Efficiency
18S	FW: GAACGAGACTCTGGGCATGC	NR_036642	102%
	REV: CTGAACGCCACTTGTCCCTC		
*DDX58*	FW: GAGCACTGGTGGATGCCTTA	XM_024996055.1	157%
	REV: GCTGTCTCTGTTGGTTCGGA		
*IRF7*	FW: GCCTCCTGGAAAACCAACTT	NM_001105040.1	127%
	REV: CCTTATGAGGGTCGGTAGGGG		
*NFKB*	FW: CGAGGTTCGGTTCTACGAGG	NM_001102101.1	134%
	REV: TGCAGGAACACGGGTTACAGG		
*IFNB*	FW: TCCAGCACATCTTCGGCATT	NM_174350.1	104%
	REV: TTCCCTAGGTGGGGAACGAT		
*ISG15*	FW: GGTATCCGAGCTGAAGCAGTT	NM_174366	115%
	REV: ACCTCCCTGCTGTCAAGGT		
*STAT4*	FW: TTCTTCCCATGTCGCCAAGT	NM_001083692.2	104%
	REV: AACCAGATGTGATTGTTGGCA		
*IFI30*	FW: GCATGCAGCTCTTGCACATC	NM_001101251.2	131%
	REV: GGCCCCAAGAGTTCTTACCC		
*PSMB9*	FW: ATCTACCTGGCCACCATCAC	NM_001034388	115%
	REV: AGGAGAGTCCGAGGAAGGAG		
*PSMB8*	FW: ACTGGAAGGCAGCACAGAGT	NM_001040480	124%
	REV: ATTGTGCTTAGTGGGGCATC		
*B2M*	FW: AFTAAGCCGCAGTGGAGGT	AC_000167.1	123%
	Rev: CGCAAAACACCCTGAAGACT		
*CXCL10*	FW: ACACCGAGGCACTACGTTCT	CB533091	121%
	Rev: TAAGCCCAGAGCTGGAAAGA		
*CXCL16*	FW: CTTGTGAGGGCAGATTGTGA	CK770974	105%
	Rev:GGTCAATAGCTGGTTAGTTGTGAA		
*CD4*	FW: GGGCAGAACGGATGTCTCAA	NM_001103225.1	110%
	REV: ATAGGTCTTCTGGAGCCGGT		
*CD8a*	FW: TACATCTGGGCTCCCTTGGT	NM_174015.1	130%
	REV: CCACAGGCCTGGGACATTTG		
*CD8b*	FW: AGCTGAGTGTGTTGATGTTCT	NM_001105344.2	93%
	REV: TTCTGAGTCACCTGGGTTGG		
*CD79b*	FW: TGATTCCCGGGCTCAACAAC	XM_002696068.6	160%
	REV: CTGCCAGATCCGGGAACAAG		

*DDX58*: DExD/H-Box Helicase 58; *IRF7*: Interferon Regulatory Factor 7; *NFKB*: Nuclear Factor Kappa-Light-Chain-Enhancer of Activated B Cells; *IFNB*: Interferon Beta; *ISG15*: Interferon Stimulated Gene 15; *STAT4*: Signal Transducer and Activator of Transcription 4; *IFI30*: IFI30 Lysosomal Thiol Reductase; *PSMB9*: Proteasome 20S Subunit Beta 9; *PSMB8*: Proteasome 20S Subunit Beta 8; *B2M*: Beta-2-Microglobulin; *CXCL10*: C-X-C Motif Chemokine Ligand 10; *CXCL*: C-X-C Motif Chemokine Ligand 16. FW: Forward primer; REV: Reverse Primer.

## Appendix A

**Table A1:** Table of genes and their expression in respective tissues. These genes were not measured in both tissues, only one tissue.

Tissue	Target	Control Mean	Control SEM	TI Mean	TI SEM	PI Mean	PI SEM	TI v. C *p* value	PI v. C *p* value
Thymus	*STAT1*	1.32	0.48	2.29	0.21	0.04	0.01	0.58	0.07
Thymus	*IFI6*	1.10	0.20	2.09	0.49	0.10	0.02	0.09	0.09
Thymus	*CXCL10*	1.24	0.35	3.10	1.20	0.27	0.12	0.99	0.11
Thymus	*CXCL16*	1.22	0.34	1.21	0.27	0.05	0.01	0.99	0.01
Thymus	*CXCR6*	1.40	0.57	1.64	0.41	0.48	0.15	0.99	0.53
Thymus	*TAP1*	1.09	0.20	1.07	0.21	0.20	0.06	0.99	0.01
Thymus	*B2M*	1.17	0.31	1.33	0.24	0.04	0.01	0.89	0.01
Thymus	*CIITA*	1.19	0.27	1.35	0.35	0.35	0.08	0.99	0.08
Spleen	*STAT4*	1.62	0.83	2.25	0.51	0.32	0.04	0.43	0.08
Spleen	*PSMB8*	1.66	0.79	6.09	1.40	0.06	0.02	0.21	0.07

SEM: Standard Error Mean; TI: Transient Infection; PI: Persistent Infection; C: Control; STAT1: Signal Transducer and Activator of Transcription 1; IFI6: Interferon Alpha Inducible Protein 6; CXCL10: C-X-C Motif Chemokine Ligand 10, CXCL16: C-X-C Motif Chemokine Ligand 16; TAP1: Transporter 1 ATP Binding Cassette Subfamily B Member; B2M: Beta-2-Microglobulin; CIITA: Class II Major Histocompatibility Complex Transactivator; STAT4: Signal Transducer and Activator of Transcription 4; PSMB8: Proteasome 20S Subunit Beta 8.
